# Identification of biomarkers for amyotrophic lateral sclerosis by comprehensive analysis of exosomal mRNAs in human cerebrospinal fluid

**DOI:** 10.1186/s12920-019-0473-z

**Published:** 2019-01-10

**Authors:** Kentaro Otake, Hidenori Kamiguchi, Yoshihiko Hirozane

**Affiliations:** Innovative Biology Laboratories, Neuroscience Drug Discovery Unit, Takeda Pharmaceutical Company Limited, 26-1, Muraoka-Higashi 2-chome, Fujisawa, Kanagawa 251-8555 Japan

**Keywords:** Extracellular vesicles, Exosomes, Cerebrospinal fluid, Liquid biopsy, Biomarker, Next-generation sequencing, Amyotrophic lateral sclerosis

## Abstract

**Background:**

Exosomes are a subset of extracellular vesicles 30–200 nm in diameter secreted from cells, which contain functional mRNAs and microRNAs. Cerebrospinal fluid (CSF) is the primary source for liquid biopsy to examine diseases in central nervous system. To date, there is no available method to analyze exosomal mRNAs comprehensively in human CSF.

**Methods:**

The main purpose of this study is to established the methodology of comprehensive analysis of exosomal mRNAs in CSF by a highly sensitive next-generation sequencing. The signatures of CSF exosomal mRNAs were then compared between four normal healthy donors and four sporadic amyotrophic lateral sclerosis patients to identify disease-related biomarkers. Differentially expressed genes were identified by DESeq2.

**Results:**

RNA sequencing from CSF exosomes was successfully performed, that was demonstrated by the high pearson’s product-moment correlation coefficient (*r* = 0.993) in the technical replicates. Also, position coverage analysis revealed that most detected mRNAs retained their integrity throughout their full-length in CSF exosomes. In CSF exosomes from normal healthy donors, an average of 14,807 genes were detected, of which 4580 genes were commonly detected among four individuals, including neuron-enriched genes such as TUBB3 and CAMK2A. In comparison with exosomal mRNAs in CSF from four patients with amyotrophic lateral sclerosis, 543 genes were significantly changed, as represented by CUEDC2. Gene Ontology analysis and pathway analysis with these genes revealed functional enrichment of ubiquitin-proteasome pathway, oxidative stress response, and unfolded protein response. These pathways are related to pathomechanisms of amyotrophic lateral sclerosis.

**Conclusion:**

We successfully established the methodology of comprehensive analysis of exosomal mRNAs in human CSF. It was shown to be useful to identify disease biomarkers for central nervous system. Several genes, such as CUEDC2, in CSF exosomes were suggested to be candidate disease biomarkers for amyotrophic lateral sclerosis.

**Electronic supplementary material:**

The online version of this article (10.1186/s12920-019-0473-z) contains supplementary material, which is available to authorized users.

## Background

Extracellular vesicles (EVs) are secreted from many types of cells to extracellular space [1]. Exosomes are known as a representative subset of EVs and are seen as cup-like shaped nanovesicles (30–200 nm) by transmission electron microscope, characterized by the presence of exosome-enriched protein markers (e.g., CD9, Flotillin-1) [2, 3]. Secretion of exosomes from the cells is considered to be dependent on the formation of multivesicular bodies preceded by sorting of cargo contents [4]. Rab GTPase and ESCRT proteins may play roles in these processes [5]. At first, exosomes were considered as a “trash box”, in which to throw away unnecessary proteins from the cells, particularly in the maturation of erythrocytes [6]. Lötvall’s group reported the presence of mRNAs and microRNAs in exosomes secreted from mast cell lines. These exosomal RNAs were transferrable and functional in recipient cells [7]. That report shed light on the exosomes as a novel mechanism of cell-to-cell communication. Exosomal mRNAs were stable against RNase and freeze-thaw process [8, 9]. These findings led to the hypothesis that exosomes play some important physiological roles. Therefore, perturbation in exosomal cargos in any disease state can be biomarkers for diagnosis, prognosis, and patient stratification. In addition, their elucidation will lead to better understanding of disease mechanisms. To date, many studies have reported specific signatures of microRNAs in plasma or serum as candidate diagnostic biomarkers [10].

In studies or examination of central nervous system, the biofluid primarily focused on is cerebrospinal fluid (CSF), as it comes into direct contact with brain tissue within the blood-brain barrier. Liquid biopsy of CSF is particularly important, as it is unfeasible to collect brain tissue from living individuals [11]. Exosomes are present in CSF and considered to reflect biological information of their source cells, especially brain tissue.

Amyotrophic lateral sclerosis (ALS) is an adult onset, fatal neurodegenerative disease characterized by the loss of motor neurons [12]. There are large unmet medical needs of disease-modifying therapy (DMT) for ALS. Indeed, nearly 50 randomized controlled trials for DMT have failed to show positive results in the past half-century. A lack of biomarkers related to the disease mechanisms is a barrier to the DMT development [13]. It is therefore highly desirable that there exist biomarkers to reflect the rate of disease progression, particularly towards implementation in clinical trials. For this reason, many efforts to identify molecular biomarkers for ALS have been made [14], for example, the protein levels of CSF neurofilament light chain and phosphorylated neurofilament heavy chain were proposed [15, 16], and recently, NAIP protein level in peripheral mononuclear cells was also reported to be correlated with the rate of disease progression [17]. Nonetheless, no specific molecular biomarker has been established for ALS. Molecular biomarkers include not only proteins, but also RNAs. Although there have been several reports on microRNA signatures in CSF as biomarkers for ALS, the studies showed inconsistent results [18–20]. Furthermore, there is no report regarding mRNAs in CSF from ALS patients. This is, at least in part, due to an insufficient detection sensitivity for exosomal mRNAs in CSF, and consequent low reproducibility. Aberrant expressions of protein-coding mRNAs are more closely linked to disease mechanisms compared with microRNAs that have post-transcriptional gene silencing effects on wide range of coding genes [21]. Therefore, an analysis of mRNAs in CSF will offer the opportunity of biomarker identification based on the disease mechanisms. However, there is no available method to analyze exosomal mRNAs comprehensively by next-generation sequencing approach in CSF exosomes.

The purpose of the current study was to establish the methodology for comprehensive analysis of exosomal mRNAs in CSF to identify disease-related biomarkers for ALS. Although ultracentrifugation is the most frequently used means to isolate EVs, there may be large technical variability in the process and it is difficult to apply for large numbers of specimens. In addition, simple and robust procedures are required for clinical settings. Therefore, we adopted the affinity spin column-based exoEasy system for the isolation of EVs. In addition, a highly sensitive RNA-seq technology was applied to those exosomal mRNAs. Here, we demonstrate the feasibility of next-generation sequencing for exosomal mRNAs (exoRNA-seq) in CSF from four normal healthy (NH) donors and sporadic ALS patients. The resultant mRNAs differentially expressed between NH and ALS enable us to speculate ALS pathomechanisms.

## Results

### Characterization of isolated EVs as exosomes

**Fig. 1:**
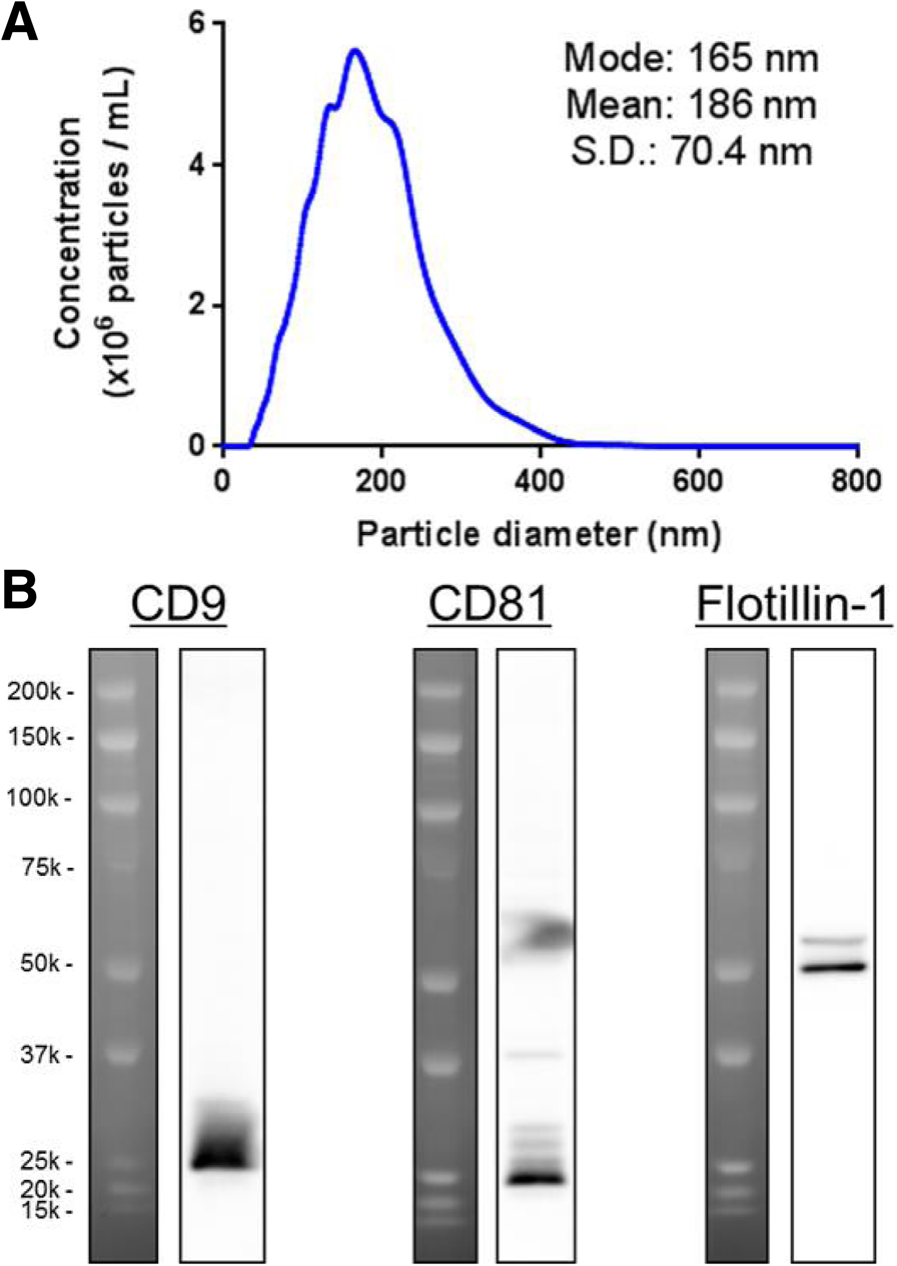
Characterization of EVs in human CSF. EVs were isolated from human CSF by exoEasy. **a** After buffer exchange to PBS, nanoparticle tracking analysis (NTA) was performed using Nanosight NS500. Mode diameter and mean diameter are described with standard deviation. **b** Presence of exosome-enriched protein markers (CD9, CD81, and Flotillin-1) were analyzed by western blotting. CD9 and CD81 corresponded to ~ 25 kDa protein band. Flotillin-1 corresponded to 48 kDa protein band

EVs from human CSF were analyzed to determine their diameters and exosome-enriched protein markers, CD9 and Flotillin-1. Nanoparticle tracking analysis (NTA) revealed that isolated EVs had a mean diameter of 186 nm ± 70.4 nm, with the highest peak, representing the most frequently measured value, at 165 nm (Fig. 1a). Furthermore, these EVs were positive for CD9, CD81, and Flotillin-1 on western blotting (Fig. 1b). CD9 and CD81 are tetraspanin proteins, whereas Flotillin-1 is an endosome-associated protein. As the EVs were concentrated by ultrafiltration using 100 kDa MWCO filters, CD9, CD81 (~ 25 kDa each), and Flotillin-1 (48 kDa) were suggested to be trapped on the filter after ultrafiltration, not as free proteins but within EVs. The presence of these proteins of different classes strongly suggested that the isolated EVs were characteristic of exosomes.

### Preparation of Illumina library for exoRNA-seq

**Fig. 2:**
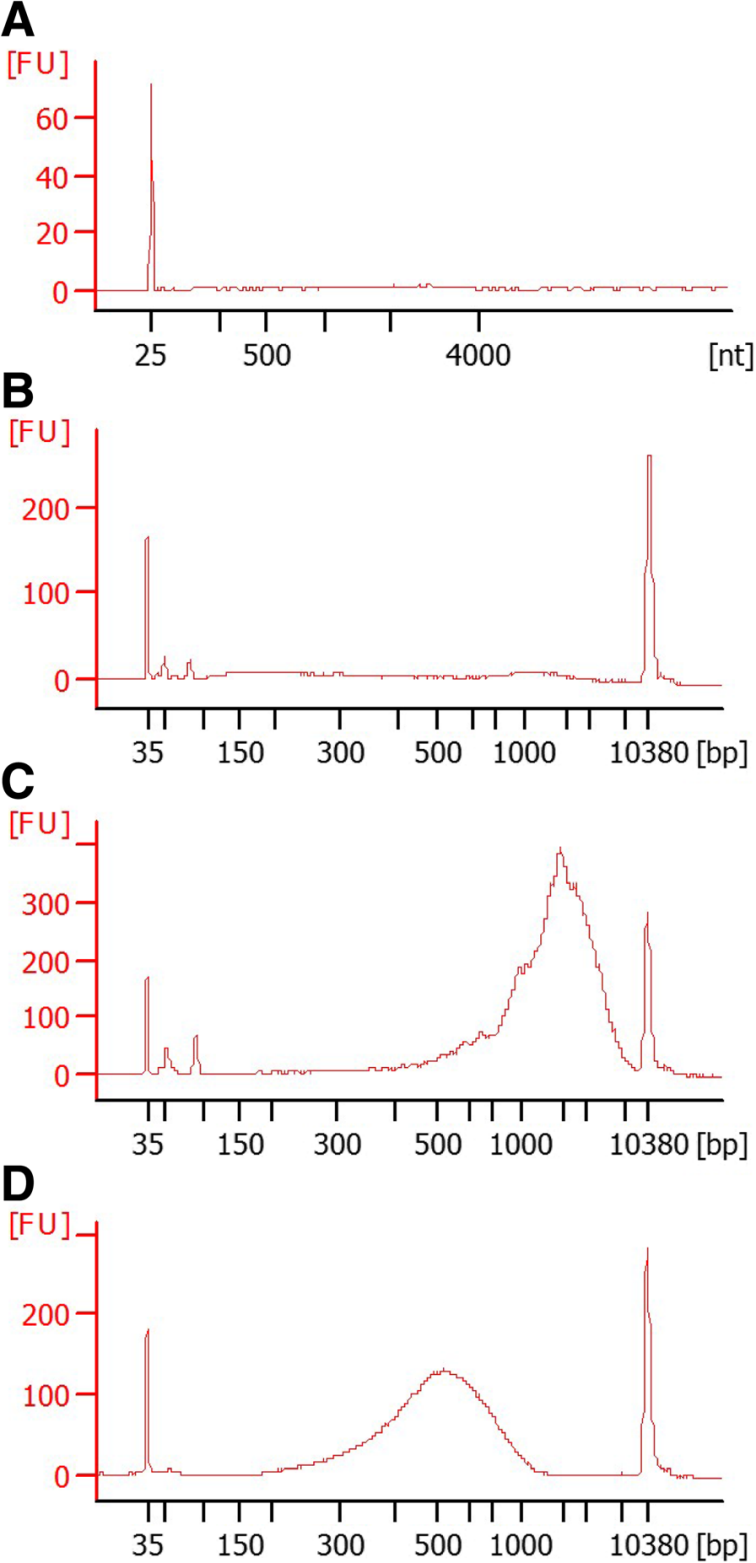
Library preparation for exoRNA-seq with ultra low input of RNA. **a** Exosomal mRNAs were purified and investigated. **b** After first-strand synthesis of cDNA from purified exosomal RNA, amplified cDNA by SMART-seq v4 was investigated. **c** As a positive control, Universal Human Reference RNA (UHR) was used. **d** Successfully prepared Illumina library for sequencing is shown

Generally, cellular total RNA or universal human reference RNA (UHR) gives prominent peaks of 18S and 28S ribosomal RNAs in the quality check test by Agilent Bioanalyzer, and RNA integrity number is calculated from this ratio. However, RNA peaks were hardly detected in exosomal RNAs purified from 930 μL of human CSF from NH donors (Fig. 2a) and ALS patients. We did not follow the RNA purification procedure provided in the exoRNeasy manufacturer’s instructions, because that method also purifies small RNAs that interfere with library preparation with ultra low input of RNA, resulting in a compromised sequencing performance. Concentrations of RNA were below or as low as the detection limit of Agilent RNA6000 pico kit (50 pg/μL) in almost all samples. To overcome this limitation, switching mechanism at 5′ end of RNA template (SMART), one of the most sensitive preparation methods for whole transcriptome amplification, was applied for the first-strand cDNA synthesis and amplification of cDNA [22]. At a glance, this scheme appeared not to succeed because there was no detectable peak in Agilent High Sensitivity DNA assay (Fig. 2b), while positive control UHR with input of 100 pg generated an amplified peak at 2500 bp (Fig. 2c). As expected, preparation of Illumina library did not turn out well with 100 pg of DNA input to Nextera XT kit, which was described in the manufacturer’s instructions. Alternatively, increase of DNA input to 1 ng successfully generated the Illumina library. The average size was approximately 500 bp, which is suitable for next-generation sequencing.

### Sequencing for exosomal mRNAs in CSF from NH donors

**Fig. 3:**
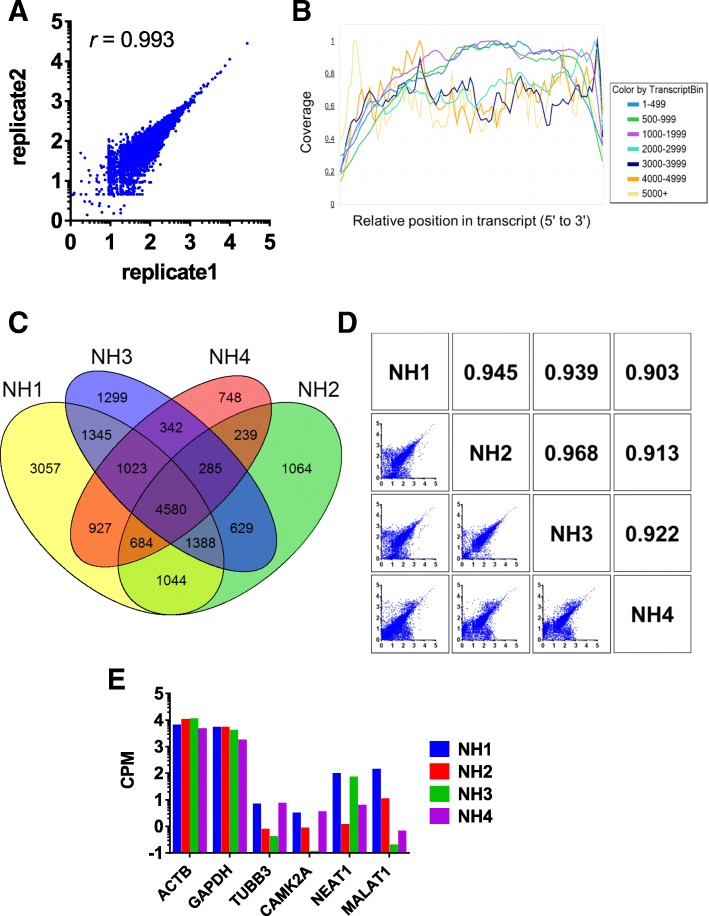
exoRNA-seq in CSF from normal healthy donors. Exosomal mRNAs in CSF from four normal healthy (NH) donors was analyzed by next-generation sequencing (exoRNA-seq). **a** To assess analytical variability, technical replicates of exoRNA-seq performed. Count per million mapped reads (CPM) of each gene was represented in log scale. *r* means pearson’s product-moment correlation coefficient. **b** Position coverage of sequence reads within overall transcript is shown. Classification of transcript bin is described based on the length of transcripts. **c** Commonly detected exosomal mRNAs in CSF from all four NH donors are visualized by Venn diagram. **d** Commonly detected 4580 genes in NH were compared among donors. CPM of each gene was represented in log scale. Pearson’s product-moment correlation coefficient was also described in each pairs of comparison. **e** Expression levels of exosomal mRNAs are shown in the scale of counts per million mapped reads (CPM)

First, we assessed the analytical variability of our exoRNA-seq. Generated reads from sequencing on MiSeq were trimmed for the Illumina adaptor sequences and mapped to the human genome reference B37.3. In the analysis of technical replicates of mRNAs from CSF exosomes, they showed the high reproducibility demonstrated by the pearson’s product-moment correlation coefficient (*r* = 0.993) in the plot by count per million mapped reads (CPM) (Fig. 3a). Next, we compared exosomal mRNAs in CSF between NH and ALS. An average of reads mapped to the human genome reference B37.3 was 37.2 million reads. An average of 14,807 genes were detected, ranging from 9814 genes to 20,598 genes (data not shown, see Additional file 1). This large variation might be partially attributed to the limited volume of CSF and extremely low input of RNA. Position coverage analysis revealed that most detected genes were covered from 5′ to 3′ end. At the same time, genes with longer transcripts showed biased coverage, possibly because of reduced efficiency in long distance-polymerase chain reaction (LD-PCR) (Fig. 3b). Considering the first-strand cDNA was primed by oligo dT primers complementary to poly-A tail, a lot of mRNAs retained their integrity throughout their full lengths. To investigate variation of contents among individuals, exosomal mRNAs in CSF exosomes from four NH donors were compared. As a result, 4580 genes were commonly detected in CSF exosomes from all four donors (Fig. 3c), accounting for 22–46% of genes detected. The abundance of these 4580 genes in each donor was compared with other three donors (Fig. 3d). Although there still existed the large variability in the genes with low expression, all pairs of comparison showed the high pearson’s product-moment correlation coefficients (*r* > 0.9). As shown in Table 1, total reads in NH4 specimens were much less than others. It might have biased effect on genes with low expression in normalization to CPM. As a result, the correlation coefficients of NH4 with other 3 NH specimens were smaller than other pairs of comparison. Commonly detected genes included cellular housekeeping genes, such as ACTB and GAPDH. These genes were abundant in the exosomes as is the case with cellular RNA (Fig. 3e). Neuron-enriched TUBB3 and CAMK2A were also detected in all samples, although in much lower amounts than ACTB. This result suggested that CSF contained exosome populations having neuronal origin. However, GFAP that is specifically expressed in astrocytes was also detected in CSF exosomes (data not shown, see Additional file 2). This meant our exoRNA-seq detected mRNA signatures not only from neuronal cells but also from other cell types in brain tissues. Not only protein-coding mRNAs, but also functional long non-coding RNAs, NEAT1 and MALAT1 [23, 24] were observed. Probably, protein-coding mRNAs and these long non-coding RNAs have different cellular trafficking from each other. Protein-coding mRNAs move to cytosol to be translated into protein, while NEAT1 and MALAT1 stay within the nuclear envelope after transcription. The mechanisms by which they are loaded to exosomes remains to be elucidated.

**Table 1:** Sample information and sequencing results

Specimen	Sex	Age	Diagnosis	Duration of disease	ALSFRS-R	Mapped reads
NH1	M	62	Healthy	N.A.	N.A.	60,623,044
NH2	M	57	Healthy	N.A.	N.A.	40,284,207
NH3	M	60	Healthy	N.A.	N.A.	54,089,758
NH4	M	56	Healthy	N.A.	N.A.	30,168,826
ALS1	M	57	sALS	1 year	45	23,718,245
ALS2	M	55	sALS	0.5 year	43	23,604,092
ALS3	M	51	sALS	5 years	42	44,024,882
ALS4	M	67	sALS	2 years	41	21,196,954

Background information on donors of cerebrospinal fluid (CSF) specimens is summarized. Additionally, read numbers of those successfully mapped to human genome reference B37.3 in exoRNA-seq are also shown. *NH* normal healthy, *sALS* sporadic ALS, N.A., represents not applicable

### Identification of differentially expressed exosomal RNAs between NH and ALS

**Fig. 4:**
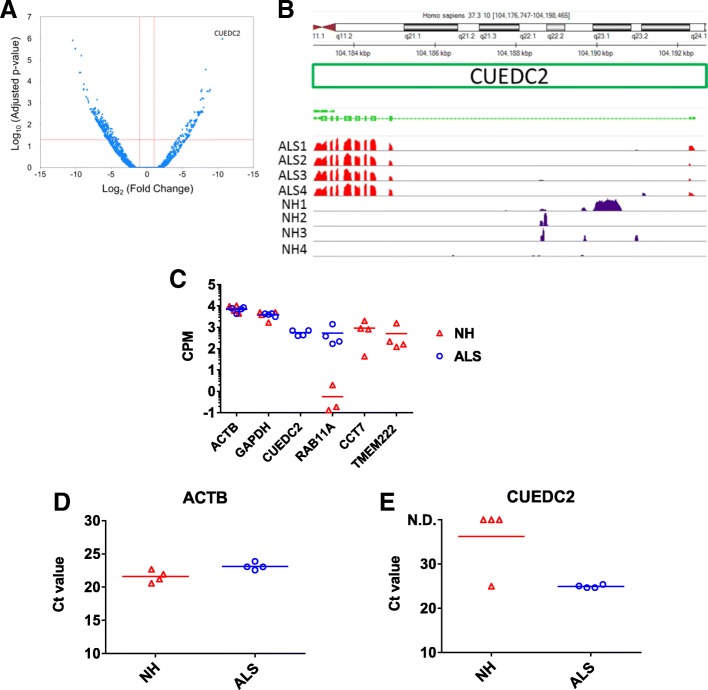
Comparison of exosomal mRNAs in CSF from NH donors and ALS patients. Differentially expressed genes (DEGs) were detected by DESeq2. **a** Volcano plot is shown with red lines (horizontal) indicating adjusted p-values less than 0.05 and (vertical) more than two fold changes in normalized count. Upper right-most plotted point represents CUEDC2. **b** Result of mapping to CUEDC2 was visualized by genome browser. Peaks in red and purple represent exon-mapped and intron-mapped reads, respectively. **c** Expression levels of DEGs and housekeeping genes were compared between NH and ALS. CPM of each gene was represented in log scale. Each point represents data for an individual. Bar represents mean value of CPM. No point on the graph means undetected. **d**, **e** Confirmatory qRT-PCR against ACTB and CUEDC2 was performed. Each point represents data for an individual. Bar represents mean value of Ct. N.D. means not detected within amplification cycle of 40

Expression profile of exosomal mRNAs in CSF from ALS patients was compared with those from NH donors, as described in the previous section. Four age- and sex-matched CSF specimens from sporadic ALS patients were chosen. These patients scored 41 to 45 on ALSFRS-R, and duration of disease ranged from six months to five years. ALS group showed significantly less total reads than NH group (Table 1). Numbers of commonly detected genes in ALS group were 3098 (cf. 4580 in NH group). Numbers of undetected genes in all four specimens were 11,719 and 13,727 in NH and ALS group, respectively. Thus, there might be some biases in the comparison between NH and ALS group. Based on the hypothesis that disease conditions in ALS would be different from healthy conditions, we did not exclude the uncommon genes in NH group in the following DESeq2 analysis. In false discovery rate (FDR) correction by Benjamini-Hochberg method, 5006 genes could be calculated for adjusted *p*-values (data not shown, see Additional file 3). Thus, the genes with no detection in any specimens or with outliers in some specimens were not calculated for adjusted p-values. DESeq2 analysis resulted in 543 genes that were significantly changed between NH donors and ALS patients groups, with adjusted p-values less than 0.05 (Fig. 4a). Among these genes, 133 genes were upregulated and 410 genes were downregulated in ALS patients group. The top 10 statistically significant DEGs are listed in Table 2. Several genes were dramatically changed between NH donors and ALS patients groups. Particularly, CUEDC2, CUE domain-containing protein-2, was only detected in ALS patients group (Fig. 4b). Interestingly, CUEDC2 has not been reported so far regarding relationship with ALS. CUEDC2 has ubiquitin-binding motif, as represented in its name, and regulates ubiquitin-proteasome pathway [25]. It is also known to be related to inflammatory response such as SOCS3 [26]. In ALS patients group, all samples covered all exon sequences of CUEDC2, suggesting that there were full-length CUEDC2 mRNAs without fragmentation in this group. However, there was no sequence mapped to this gene in NH donors group. Again, since reverse transcription relied on the presence of poly-A tail, there were two possibilities. One is that there were CUEDC2 mRNAs without in 3′ end region in NH group. Exosomes have also been considered to contain fragmented mRNAs. The other is that there was no detectable amount of the gene transcript. Next, representative DEGs were compared between the two groups. In contrast, ACTB and GAPDH, described in Fig. 3, were again depicted. ACTB and GAPDH resulted in comparable CPM in both groups (Fig. 4 and Additional file 2). As with CUEDC2, RAB11A was highly presented in ALS patients group, while low CPM was also detected in NH group. RAB11A is a member of the small GTPase superfamily, and plays roles in the transport of proteins, lipids, and vesicles. In contrast, CCT7 and TMEM222 were only detected in NH donors group. CCT7 encodes a molecular chaperone that plays roles in protein folding. The result of RNA-seq should be validated by other detection method. The abundance of ACTB and CUEDC2 in exosomal mRNAs was examined by probe-based quantitative real time polymerase chain reaction (qRT-PCR). As observed in exoRNA-seq, ACTB showed comparative level among samples although Ct values of ACTB were slightly larger in ALS group than those of NH group (Fig. 4d). It might be correlated with the reduced total reads in RNA-seq in ALS group compared with NH group. CUEDC2 could be detected in all ALS specimens and it could not be detected in NH group with exception of one specimens with detectable CUEDC2 (Fig. 4e). The difference of Ct values in CUEDC2 between NH and ALS groups gave *p*-value of 0.0569 by Welch’s t-test after confirmation of the Gaussian distribution by Kolmogorov-Smirnov test. Thus, although the statistically significant difference in CUDEC2 was not observed between NH and ALS groups, a similar expression pattern was observed in qRT-PCR.

**Table 2:** Top 10 statistically significant DEGs

UP			DOWN		
Gene	log_2_FC	FDR (BH)	Gene	Log_2_FC	FDR (BH)
CUEDC2	10.6106	1.0473E-06	CCT7	−10.4733	1.23E-06
RAB11A	8.2863	0.00003	TMEM222	−10.1012	2.99E-06
GGA2	7.7439	0.0002	HMGCL	−9.2656	6.36E-06
PPP1R16A	8.8835	0.0002	PKM	−9.4769	3.9E-05
DTNB	7.6439	0.0003	SSBP4	−9.3553	3.9E-05
RALGDS	8.4936	0.0003	THNSL2	−8.8952	0.0001
SCOC	8.7621	0.0003	HPS3	−8.9111	0.0002
TFG	8.738	0.0003	BRIX1	−8.4127	0.0002
SHB	7.7938	0.0006	UAP1	−8.9189	0.0002
AGPAT6	7.9443	0.0006	FBXL5	−8.5617	0.0005

Differentially expressed genes (DEGs) were detected by DESeq2. The top 10 statistically significant DEGs are listed with log2 fold change to NH control and adjusted *p*-values for false discovery rate (FDR) correction

To investigate whether there was functional enrichment in DEGs, GO analysis was performed. Decrease in ligase activity was ranked at the top (Table 3). Subsequently, decreases in response to oxidative stress and ubiquitin-protein ligase activity were included. Next, pathway analysis was also performed, by IPA. As in GO analysis, protein ubiquitination pathway and superoxide radical degradation, which are related to ALS pathomechanisms, were listed (Table 4). In regards to oxidative stress, mutation in SOD1 gene is the second major cause of familial ALS next to C9orf72 repeat sequence in untranslated region [27]. Although the amount of SOD1 transcript was not different between NH donors and ALS patients groups, SOD2 and SOD3 were detected as DEGs. Additionally, unfolded protein response was also specified.

**Table 3:** Top 15 GOs by BaseSpace Correlation Engine

Biogroups	direction	*P*-value
ligase activity	down	1.60E-09
ligase activity, forming carbon-nitrogen bonds	down	2.30E-09
acid-amino acid ligase activity	down	4.40E-09
kinase binding	down	5.10E-09
response to oxidative stress	down	5.90E-09
mitochondrial inner membrane	down	1.40E-07
cellular component biogenesis at cellular level	down	3.30E-07
DNA catabolic process	down	4.80E-07
protein binding transcription factor activity	down	6.90E-07
transcription cofactor activity	down	1.30E-06
ubiquitin-protein ligase activity	down	1.50E-06
response to hypoxia	down	1.90E-06
response to decreased oxygen levels	down	2.10E-06
transcription factor binding transcription factor activity	down	2.20E-06
negative regulation of cysteine-type endopeptidase activity	down	2.80E-06

Enrichment of Gene Ontology (GO) in DEGs was analyzed by BaseSpace Correlation Engine. The top 15 ranked biogroups are listed

**Table 4:** Top 15 canonical pathways by IPA

Canonical pathways	*P*-value	Genes
Sirtuin Signaling Pathway	0.0002	PPARG,POLR1D,ATG12,TIMM44,ATG10, TIMM17B,MT-ND4,GLUD1,HIF1A,ESRRA, STAT3,MT-ND4L,AGTRAP,XPA,SOD3, SOD2,MT-ND3,GOT2
Leucine Degradation I	0.0009	AUH,IVD,HMGCL
L-cysteine Degradation I	0.0031	CDO1,GOT2
Pyridoxal 5′-phosphate Salvage Pathway	0.0038	PAK1,SGK1,GRK6,GRK5,PKN1,IRAK1
Adipogenesis pathway	0.0039	PPARG,FOXC2,BMP4,HDAC11,CEBPA, XBP1,GTF2H5,SOX9,HIF1A
IL-8 Signaling	0.0062	RAB11FIP2,GNAI3,ITGB2,CCND2,RND3, RHOD,MMP2,CHUK,LASP1,GNG12,IRAK1
Salvage Pathways of Pyrimidine Ribonucleotides	0.0065	NME3,PAK1,SGK1,GRK6,GRK5,PKN1, IRAK1
RhoGDI Signaling	0.0081	GNAI3,PAK1,PPP1R12C,ARHGAP9,RND3, PAK6,RHOD,CD44,GNG12,MSN
Unfolded protein response	0.0085	PPARG,PDIA6,ERO1B,CEBPA,XBP1
Protein Ubiquitination Pathway	0.0087	PSMB4,UBE2G2,USP18,USP14,USP4, UCHL5,USP19,PSMD14,DNAJA1,UBE2D1, UBE2L6,UCHL3,UBE2J2
Superoxide Radicals Degradation	0.0135	SOD2,SOD3
Pyrimidine Ribonucleotides Interconversion	0.0195	NUDT5,NME3,ENTPD1,CTPS1
Ketogenesis	0.0209	BDH2,HMGCL
Pyrimidine Ribonucleotides De Novo Biosynthesis	0.0229	NUDT5,NME3,ENTPD1,CTPS1
Dolichyl-diphosphooligosaccharide Biosynthesis	0.0251	DPM1,ALG8

Enrichment of canonical pathway in DEGs was analyzed by Ingenuity Pathway Analysis (IPA). The top 15 ranked pathways are listed

## Discussion

In this study, we achieved to develop exoRNA-seq that could identify DEGs in CSF between ALS and NH groups. Also, this is the first report to show the integrity of exosomal mRNAs in CSF. EVs in CSF were isolated using spin column-based exoRNeasy technology instead of ultracentrifugation. The validity of exoRNeasy was previously reported in comparison with ultracentrifugation for RNA analysis in serum and plasma EVs [28]. We demonstrated that EVs isolated from human CSF were also characterized as exosomes. In addition to our finding here that neuron-enriched genes were present in CSF exosomes, there was a report that repertoire of exosomal microRNAs in human CSF reflected those in brain tissues [29]. Although it is difficult to prove physiological roles of these exosomal mRNAs and microRNAs in adult humans, exosomes secreted from neurons are considered to play a role in synaptic activity [30]. Thus, exosomes in CSF are thought to be a useful source for investigation of alteration in neuronal cells as a surrogate material of brain. Besides traditional proteomic or microRNA profiling analysis, our exoRNA-seq provides the unique and novel approach to methodologies of biomarker identification. We reported that more than 4500 genes were commonly detected in exosomes in CSF from NH donors, and also showed that housekeeping genes such as ACTB and GAPDH in the exosomes were also abundant and consistent among four individuals. DESeq2 can be applied to RNA-seq with normalization by logarithm of geometric mean of all genes. Upon further validation in RT-PCR, how the data is normalized should be considered because there is no information in housekeeping genes in exosomes. If ACTB and GAPDH were to show stable amounts among individuals in a large cohort, they could be used as internal controls, as is the case with cellular RNAs.

We demonstrated that exosomal mRNAs can feasibly be biomarkers for diagnosis of ALS. CUEDC2 was the most dramatically increased exosomal mRNA in CSF from ALS patients. At least, inflammatory cytokines such as IL-6 were elevated in CSF from ALS patients compared with control donors and patients with multiple sclerosis [31]. The increase in CUEDC2 might reflect neuroinflammation related to the regulation of SOCS3. Also, ubiquitin-proteasome pathway is related to pathomechanisms of ALS. In lesions in ALS, ubiquitin-immunoreactive inclusion bodies were observed [32]. TDP-43 protein coded by TARDBP gene was found to co-localize in these inclusion bodies [33]. In pathologic condition, TDP-43 is considered to be ubiquitinated, truncated, phosphorylated, and misfolded [34]. The exact mechanisms of increase in exosomal CUEDC2 remains to be elucidated. Molecular chaperone-related CCT7 was also related to protein folding in cellular model of ALS [35]. Ubiquitin-proteasome pathway and unfolded protein response in pathway analysis may reflect these aspects of pathomechanisms. Oxidative stress response is also related to ALS pathomechanisms. Top-ranked sirtuin signaling pathway is also related to oxidative stress response. It was also reported that SIRT1 was upregulated in mouse model of ALS [36].

As this study focused on the technical development of exoRNA-seq and feasibility of biomarker identification by exoRNA-seq, it was performed with small sample size of four NH donors and four ALS patients. There existed two limitations in this study. One is low statistical power due to small sample size. It was considered as a main reason why so many genes were determined as DEGs. In DESeq2, FDR correction can be applied to the genes without no outlier identified cook’s distance. Small sample size might not be enough to identify true outliers. The other is extremely low abundance of exosomal mRNAs that might cause the large variation of gene expression among four NH specimens in Fig. 3d. However, the reproducibility of our method was assured as shown in Fig. 3a. Thus, the large variation among four NH samples was not caused by technical instability but by another factors such as biological variation or artificial characteristics of specimens (i.e. Preservation period of the commercially available CSF after collection was different from each other.). The low abundance of exosomal mRNAs was observed especially in ALS. In comparison between NH and ALS group, there were more downregulated genes than upregulated genes. This is possibly due to some biases in genes with low expression upon normalization process. However, it was notable that upregulated genes were also detected in exoRNA-seq and same tendency was confirmed by qRT-PCR in respect to CUEDC2. Consequently, we identified biomarker candidates that were linked to pathomechanisms of ALS. The significance of these candidate genes should be verified with large sample size that ensure enough statistical power. Specificity among other neurodegenerative diseases is also important and should be verified.

## Conclusions

We successfully developed and implemented exoRNA-seq in CSF. We found exosomal mRNAs in CSF as a promising biomarker to reflect pathomechanisms of ALS. Thus, we conclude that comprehensive analysis of exosomal mRNAs in CSF is useful to identify candidates of disease biomarkers for central nervous system with speculation of disease pathomechnism.

## Methods

### CSF specimens

CSF specimens used for set-up of exosome characterization were purchased from Science Care (AZ, USA) or Proteogenex (CA, USA). CSF specimens from four NH donors and four ALS patients matched by age and sex were purchased from PrecisionMed (CA, USA). All donors for the specimens in this study provided informed consent. Detailed information on specimens such as age, sex, duration of disease, and ALS Functional Rating Scale-Revised (ALSFRS-R) is shown in Table 1. CSF specimens were stored at − 80 °C until use. All experiments were performed under approval of the ethical review board of our institution.

### Purification of exosomal RNA or intact EVs from CSF

CSF specimens up to 1 mL were thawed on ice. Residual dead cells were removed by centrifugation at 2000 x g, 4 °C for 5 min. Cell debris that included large EVs (apoptotic body, microvesicle) was further removed by centrifugation at 10,000 x g, 4 °C for 20 min. Approximately 930 μL of supernatant was transferred to new Protein-LoBind Tubes (Eppendorf, Hamburg, Germany) and mixed with equal volume of Buffer XBP in exoRNeasy Serum/Plasma Midi Kit (QIAGEN, Hilden, Germany). The mixture was immediately applied on exoRNeasy midi column and centrifuged at 500 x g, room temperature (RT) for 1 min. The column was washed once with 3.5 mL of Buffer XWP and centrifuged at 3000 x g, RT for 5 min. RNA from trapped EVs on the column was extracted by Proteinase K containing lysis buffer provided in Agencourt RNAdvance Tissue Kit (Beckman Coulter, CA, USA). After on-column disruption of EVs, the lysate was recovered to collection tube by centrifugation at 3000 x g, RT for 5 min. Following procedures were performed in accordance with the instructions provided with Agencourt RNAdvance tissue kit, except for final elution volume of 25 μL. Purified RNA was investigated using Agilent RNA6000 pico kit on 2100 Bioanalyzer (Agilent Technologies, CA, USA).

To purify intact EVs, samples on the column were eluted by XE buffer in exoEasy Maxi Kit (QIAGEN) without RNA extraction.

### NTA

For NTA, purified EVs were concentrated and dispersion medium was exchanged with phosphate-buffered saline by Vivacon-500,100 kDa molecular weight cutoff (MWCO) spin column (Sartorius, Gottingen, Germany). Presence of nanoparticles and their Brownian motion were observed using a Nanosight NS500 (Malvern Instruments, Malvern, UK). Particle diameter was calculated from velocity of Brownian motion.

### Western blotting

Intact EVs were isolated and concentrated as described in the procedure for NTA. They were mixed with 4x NuPAGE LDS sample buffer and 10x Sample Reducing Agent (Thermo Fisher Scientific, MA, USA). The mixture was incubated at 70 °C for 10 min for reduction of disulfide bond. Proteins were separated by electrophoresis with NuPAGE SDS-PAGE system (Thermo Fisher Scientific) with protein size standard (Bio-Rad Laboratories, CA, USA) and blotted to PVDF membrane (Bio-Rad Laboratories). The blot was blocked with PVDF Blocking Reagent for Can Get Signal (TOYOBO, Osaka, Japan). To identify isolated EVs as exosomes, primary antibodies against exosome-enriched protein markers CD9, CD81 (mouse mAb from Novus Biologicals, CO, USA), and Flotillin-1 (mouse mAb from BD Biosciences, NJ, USA) were used. All of them were diluted 1000-fold in Can Get Signal solution 1. HRP-conjugated anti-mouse secondary antibodies (Cell Signaling Technology, MA, USA) were used with dilution of 5000-fold in Can Get Signal solution 2.

### Library preparation for exo-mRNA-seq

To prepare a library for exo-mRNA-seq from extremely low amount of RNA, SMART-seq v4 Ultra Low Input RNA Kit for Sequencing was used (Takara Bio, Shiga, Japan). UHR was used as a positive control (Agilent Technologies). Input volume of RNA was adjusted to 9.5 μL, which is the maximal volume described in the instructions. Amplification of cDNA was performed for 15 cycles for LD-PCR. The concentration of purified cDNA was measured with Qubit dsDNA HS Kit (Thermo Fisher Scientific). Illumina sequencing adaptors and indices were ligated to tagmented DNA by Nextera XT DNA Library Prep Kit (Illumina, CA, USA) with input DNA amount of 1 ng. Size distribution and concentration of prepared library was evaluated using an Agilent High Sensitivity DNA Kit (Agilent Technologies) and Qubit dsDNA HS Kit, respectively.

### Next-generation sequencing

Paired-end sequencing for 76 bp was run on MiSeq and NextSeq 500 system with MiSeq Reagent Kit v3 and NextSeq 500/550 High Output Kit, respectively (Illumina). The run for technical replicates of mRNAs from CSF exosomes was performed on MiSeq. ExoRNA-seq for the comparison between NH and ALS was run on NextSeq 500. Each read in generated FASTQ file was mapped to human genome reference B37.3 with the aid of RNA-seq pipeline implemented in OmicSoft ArrayStudio (QIAGEN). Relative expression of exosomal mRNAs was calculated as CPM. Venn diagram was constructed using VennDiagram package implemented in R (distributed by CRAN) under operation of its integrated development environment RStudio.

### Statistical analysis

To identify differentially expressed genes (DEGs) in exosomal mRNAs, row count data was normalized by logarithm of geometric mean and size factor was calculated. DEGs were detected using DESeq2 function in Array Studio in which adjusted *p*-values for FDR were calculated by Benjamini-Hochberg method with alpha level of 0.05. The presence of the Gaussian distribution in Ct values generated from qRT-PCR was examined by Kolmogorov-Smirnov test. The difference of an average of Ct values generated from qRT-PCR was examined by Welch’s t-test. Typical DEG was confirmed in genome browser for the mapped reads. Gene Ontology (GO) analysis was performed using BaceSpace Correlation Engine database (Illumina). Pathway analysis was performed using Ingenuity Pathway Analysis (IPA) (QIAGEN).

### qRT-PCR

To confirm DEGs detected in exoRNA-seq, probe-based qRT-PCR was performed to examine ACTB and CUEDC2. Mixtures of primers and probes were synthesized and purchased from Integrated DNA technologies (IL, USA). The sequences were as follows: ACTB forward (5’-CCTTGCACATGCCGGAG-3′)/reverse (5’-ACAGAGCCTCGCCTTTG-3′)/probe (/56-FAM/TCATCCATG/ZEN/GTGAGCTGGCGG/3IABkFQ/), CUDEC2 forward (5’-CGACGGAGCAGAAGAGAGA-3′)/reverse (5’-GAGGTGTGTCTGGACAAAGG-3′)/probe (/56-FAM/ATGGAGCTG/ZEN/GAGAGGATCGTCAGT/3IABkFQ/). Pre-amplification of cDNA was performed with TaqMan PreAmp Master Mix (Thermo Fischer Scientific) in accordance with the instruction of manufacturer. Mixture of primers and probes, TaqPath qPCR Master Mix (Thermo Fischer Scientific), and pre-amplified cDNA was mixed and qRT-PCR was performed on Applied Biosystems 7900HT Fast Real-Time PCR System (Thermo Fischer Scientific). Ct value was calculated from amplification curve with threshold of 0.2 and automatic baseline condition.

## Additional files

Additional file 1: Mapping profile of exoRNA-seq. Mapping profile of exoRNA-seq reads to human genome reference B37.3 are summarized. (XLSX 11 kb)

Additional file 2: CPM in exoRNA-seq. CPM values for all genes and specimens generated from exoRNA-seq are listed. (XLSX 2194 kb)

Additional file 3: Results of DESeq2. Logarithm of fold-change, raw *p*-value, and adjusted *p*-value after FDR correction are listed. (XLSX 682 kb)

## Abbreviations

ALS: Amyotrophic lateral sclerosis
ALSFRS-R: ALS Functional Rating Scale-Revised
CPM: Counts per million mapped reads
CSF: Cerebrospinal fluid
EVs: Extracellular vesicles
exoRNA-seq: Exosomal messenger RNA sequencing
FDR: False discovery rate
GO: Gene Ontology
LD-PCR: Long distance polymerase chain reaction
NTA: Nanoparticle tracking analysis
qRT-PCR: Quantitative real time polymerase chain reaction
RT: Room temperature
SMART: Switching mechanism at 5′ end of RNA template
UHR: Universal human reference RNA
